# Prosapogenin A induces apoptosis in human cancer cells *in vitro* via inhibition of the STAT3 signaling pathway and glycolysis

**DOI:** 10.3892/ol.2013.1561

**Published:** 2013-09-04

**Authors:** TIAN-XIAO WANG, ZHONG-QING ZHANG, YUE CONG, XIAO-YAN SHI, YING-HUA LIU, FANG-LI ZHAO

**Affiliations:** Institute of Traditional Chinese Medicine, College of Pharmacy, Henan University, Kaifeng, Henan 475004, P.R. China

**Keywords:** prosapogenin A, apoptosis, STAT3, glycolysis

## Abstract

Signal transducer and activator of transcription 3 (STAT3) is considered to be an oncogene. Blocking STAT3 signaling may induce growth arrest and apoptosis in different types of tumors. Cancer cells utilize the glycolytic pathway to maintain cell growth even when adequate oxygen is present. Glycolysis inhibition is a potential therapeutic modality. In the present study, the effects of Prosapogenin A (PSA) from the traditional Chinese medicine, *Veratrum*, on apoptosis, the STAT3 signaling pathway and glycometabolism in cancer cells were investigated. The results indicated that PSA induced growth inhibition and apoptosis in HeLa, HepG2 and MCF-7 cells. PSA inhibited the STAT3 signaling pathway and modulated the expression of glycometabolism-related genes. The results indicate that the inhibition of the STAT3 signaling and glycometabolism pathways contributes to the PSA-mediated apoptosis of HeLa, HepG2 and MCF-7 cells.

## Introduction

Signal transducers and activators of transcription (STAT) proteins are transcription factors that regulate critical cell functions. STAT3, in particular, is constitutively active in a wide variety of human cancer cells and plays significant roles cancer cell growth (1–6). STAT3 has the ability to promote malignancy, and deficiency in STAT3 leads to the resistance to transformation. Thus, STAT3 is considered to be an oncogene (7–10). Blocking STAT3 signaling through STAT3 antisense oligonucleotides or RNA interference may induce growth arrest and apoptosis in various tumor types. Blocking STAT3 is neither harmful nor toxic to normal cells (11–13), providing further evidence for the potential of STAT3 as a target for cancer treatment.

Numerous efforts have been made to identify effective agents against cancer, particularly from herbal medicines. In China, traditional Chinese medicine has been widely used in cancer therapy, and has been shown to effectively control cancer progression and prolong survival times in cancer patients. Based on the principles of traditional Chinese medicine and the progress in the pharmacological characterization of anticancer herbs, we developed a saponin from *Veratrum* (Prosapogenin A) for the treatment of cancer.

*Veratrum* is a type of traditional Chinese medicine. Previous research has shown that *Veratrum* significantly inhibits the growth of human leukemia HL-60, human gastric carcinoma BGC-823, human liver carcinoma BEL-7402 and human nasopharyngeal carcinoma KB (14–16) cells.

In the present study, it was observed that PSA from *Veratrum* inhibited proliferation and induced apoptosis in human cervical carcinoma (HeLa), hepatocellular carcinoma (HepG2) and breast adenocarcinoma (MCF-7) cells. PSA induced cell cycle arrest in the G_2_/M phase in the HepG2 cells, together with the downregulation of STAT3 expression and the modulation of STAT3 downstream genes. PSA also modulated the expression of glycometabolism-related genes.

## Materials and methods

### Materials and reagents

RPMI-1640, fetal bovine serum (FBS), penicillin-streptomycin, trypsin-EDTA and TRIzol reagent were purchased from Invitrogen Life Technologies (Carlsbad, CA, USA). MTT, Hoechst 33342 and propidium iodide (PI) were obtained from Sigma (St. Louis, MO, USA). The RT-PCR kit was provided by Promega (Madison, WI, USA). STAT3, pSTAT3, β-actin primary antibodies and horseradish peroxidase (HRP)-conjugated secondary antibodies were obtained from Cell Signaling Technology, Inc. (Danvers, MA, USA). A Human STAT3-Regulated cDNA Plate Array kit (AP-0151) was purchased from Signosis, Inc. (Santa Clara, CA, USA). PSA was extracted from *Veratrum* using 70% ethanol and then separated, purified and characterized.

### Cell culture and cell viability

Cell lines were obtained from the American Type Culture Collection (Manassas, VA, USA). Human cervical carcinoma (HeLa), hepatocellular carcinoma (HepG2), breast adenocarcinoma (MCF-7) and human non-cancer 7701 and 293 cells were cultured in RPMI-1640 supplemented with 10% FBS and penicillin/streptomycin (100 U/ml) at 37°C in an atmosphere of 5% CO_2_. The cells were exposed to PSA of varying concentrations (0–15 μM) for 24, 48 and 72 h. The ability to reduce survival was assessed as IC_50_ values. Ginsenoside Rh2 was used as a positive control.

### Cell apoptosis and cell cycle analysis

Cell apoptosis was studied by double staining with Hoechst 33342 and PI and using Array Scan VTIHCS600 High-Contents (Thermo Scientific, Waltham, MA, USA). The cells were incubated in 96-well plates in either the presence (5 or 10 μM) or absence of PSA for 48 h. The cells were then incubated with 5 mg/l Hoechst 33342 for 10 min and 5 mg/l PI for another 1 h in the dark, followed by washing twice with ice-cold PBS and subjection to Array Scan VTIHCS600 High-Contents to record the red fluorescence produced by the PI. The cells treated with Rh2 were used as a positive control.

To analyze the cell cycle profile, cells treated with PSA (0, 5 or 10 μM) for 48 h were fixed with 70% ethanol at 4°C for 12 h and then stained in PI solution (50 μg/ml PI, 100 μg/ml RNase A and 0.2% v/v Triton X-100) for 20 min at 37°C. The stained cells were then subjected to DNA content analysis by flow cytometric analysis using FACScalibur flow cytometer (BD Biosciences, San Jose, CA, USA). and the data obtained were processed using Summit software.

### RNA extraction and RT-PCR analysis

Total RNA was isolated from the cancer cells using TRIzol reagent. Oligo(dT)-primed RNA (1 μg) was reverse-transcribed and the obtained cDNA was used to determine the mRNA quantity of STAT3, using the RT-PCR kit according to the manufacturer’s instructions. β-actin was used as an internal control. The primers used for amplification of the STAT3 and β-actin transcripts are as follows: STAT3 forward, 5′-CCAAGGAGGAGGCATTCG-3′ and reverse, 5′-ACATCGGCAGGTCAATGG-3′; size, 147 bp; and β-actin forward, 5′-CTTCTACAATGAGCTGCGTG-3′ and reverse, 5′-TCATGAGGTAGTCAGTCAGG-3′; size, 305 bp. The expression of the glycometabolism-related genes was also detected by RT-PCR: GLUT1 forward, 5′-CAACGCTGTCTTCTATTACTC-3′ and reverse, 5′-GCCACGATGCTCAGATAG-3′; size, 252 bp; HK forward, 5′-CCAGAAGGCTCAGAAGTC-3′ and reverse, 5′-ATGCTTGTCCAGGAAGTC-3′; size, 216 bp; PFKL forward, 5′-TCCGCATCTATGGTATTCAC-3′ and reverse, 5′-GTCTTCATC TTCTCCGTCAT-3′; size, 400 bp.

### Western blotting

The tumor cells were lysed by RIPA and centrifuged at 12,000 × g for 10 min, followed by determination of the protein concentration in the supernatants. Equal amounts of protein per lysate were separated by SDS-PAGE and transferred to polyvinylidene difluoride membranes, followed by blocking in Tris-buffered saline (TBS) containing skimmed dry milk (3% w/v). The membranes were incubated with the primary rabbit anti-human STAT3, pSTAT3 and β-actin monoclonal antibodies (Cell Signaling Technology, Inc.) diluted to 1:1,000 in PBS at 4°C overnight, then incubated with the HRP-conjugated secondary antibody (goat anti-rabbit IgG, Cell Signaling Technology, Inc.) This was followed by enhanced chemiluminescence detection.

### STAT3-regulated downstream gene cDNA assay

STAT3 mediates the expression of its target genes, which are involved in a diverse array of biological processes, including oncogenesis, cell growth, differentiation and apoptosis. A STAT3-regulated cDNA plate array (Signosis, Inc.) was used to detect the expression of the STAT3 target genes. The cDNA plate array is a plate-based hybridization profiling analysis for monitoring the expression of dozens of genes through the reverse transcription of mRNA into cDNA. Like array analyses, total RNA is first reverse-transcribed into cDNA in the presence of biotin-dUTP in the assay. Targeted genes are then specifically captured onto individual wells on a plate through a pre-coated gene-specific oligonucleotide. The captured cDNAs are further detected with streptavidin-HRP. Luminescence is reported as relative light units on a microplate luminometer. The expression level of the genes is directly proportional to the luminescence intensity.

### Statistical analysis

All data were expressed as the mean ± standard deviation. Statistical differences between groups were analyzed by one-way ANOVA then a post-hoc t-test. P<0.05 was considered to indicate a statistically significant difference.

## Results

### Effect of PSA on cell viability

**Figure 1:**
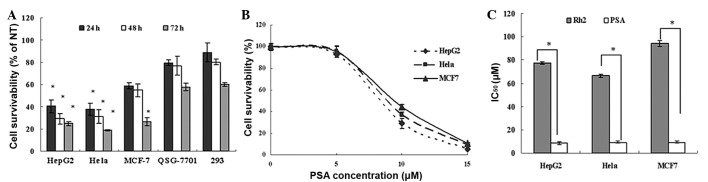
PSA significantly affects cancer cell survival. (A) The effect of PSA treatment (10 μM, 24, 48 and 72 h) on a range of cancer cells and non-cancer cells as determined by MTT assay. Results show the percentage of cell viability to non-treatment (NT) and are represented as the mean ± SD. n=3 (^*^P<0.01). (B) Concentration-response curves showing the effect of PSA treatment for 48 h on the survival of HepG2, HeLa and MCF-7 cells. Results show the mean ± SD. n=3. (C) IC_50_ of HepG2, HeLa and MCF-7 cells treated with PSA and positive control Rh2 for 48 h. Results show the mean ± SD. n=3. (^*^P<0.01). PSA, Prosapogenin A.

To determine the effect of PSA and the positive control Rh2 on a wider panel of human cancer cell lines, the cell survival of three cancer cell lines of different origins [cervical carcinoma (HeLa), hepatocellular carcinoma (Hep G2) and breast adenocarcinoma (MCF-7) cells] was determined in comparison with that of normal human hepatocyte 7701 and 293 cells. The effect of 10 μM PSA treatment for 24, 48 and 72 h on a range of cancer and non-cancer cells was determined by MTT assay. The results showed that 10 μM PSA significantly inhibited the growth of the cancer cells in a time-dependent manner. Notably, PSA did not significantly affect the survival of the human non-cancer 7701 and 293 (Fig. 1A) cells. The concentration-response correlation between PSA (0–15 μM) and reduced cell survival was also examined in the HeLa, HepG2 and MCF-7 cells. PSA significantly inhibited the growth of the cancer cells in a concentration-dependent manner (Fig. 1B). The IC_50_ values for this compound were 8.41, 9.36 and 9.27 μM (all n=3) in the HepG2, MCF7 and HeLa cells, respectively, and were significantly lower in comparison with those for the positive control, Rh2 (Fig. 1C).

### Apoptosis and cell cycle analysis

**Figure 2:**
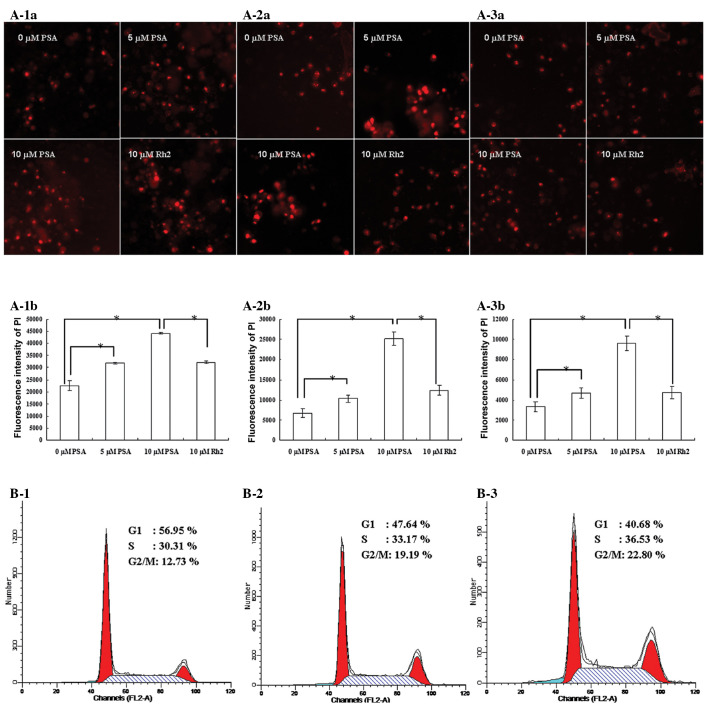
PSA induces cell apoptosis and cell cycle arrest. (A) Effect of PSA on cell apoptosis. Cells treated with 0, 5 and 10 μM of PSA and 10 μM of Rh2 for 48 h were incubated with 5 mg/l Hoechst 33342 for 10 min and 5 mg/l PI for another 1 h in the dark, then MFI of PI were detected by Array Scan VTIHCS600 High-Contents. (A-1-a): Apoptosis image of Hela cells. (A-2-a) Apoptosis image of HepG2 cells. (A-3-a) Apoptosis image of MCF7 cells. (A-1-b) MFI of PI in HeLa cells. (A-2-b) MFI of PI in HepG2 cells. (A-3-b) MFI of PI in MCF7 cells (^*^P<0.05). (B) PSA-induced cell cycle arrest detected by flow cytometry. (B-1) HepG2 cells treated with 0 μM PSA; (B-2) HepG2 cells treated with 5 μM PSA; (B-3) HepG2 cells treated with 10 μM PSA. Insets show percentage distribution of cells in each cell cycle phase. PSA, Prosapogenin A; PI, propidium iodide; MFI, mean fluorescence intensity.

Apoptosis is programmed cell death that is characterized by specific structural changes. PI and Hoechst 33342 double staining was used to detect the changes of apoptotic cells treated with PSA and Rh2. The apoptosis-inducing effects of PSA were assessed by Array Scan VTIHCS600 High-Contents. All three cancer cell lines treated with 5 and 10 μM PSA for 48 h showed a significant increase in apoptotic bodies compared with that in the untreated group. By comparison, the apoptotic cell count in the groups treated with 10 μM Rh2 was significantly lower compared with that in the groups treated with 10 μM PSA (Fig. 2A).

The effect of PSA on the cells was also examined using a cell cycle analysis. The effect of PSA on cell cycle activity was analyzed by performing PI staining followed by detection with flow cytometry. PSA induced cell cycle arrest in the HepG2 cells in the G_2_/M phase (Fig. 2B). There was a significant accumulation of cells in the G_2_/M phase for those cells treated with 5 and 10 μM of PSA (19.19 and 22.80%, respectively) compared with the untreated cells (12.73%). This indicates that there is a blockage in the G_2_/M phase, which may cause cell growth suppression or apoptosis by inhibiting the mitosis of cells.

### PSA inhibits the expression of STAT3 mRNA and pSTAT3

**Figure 3:**
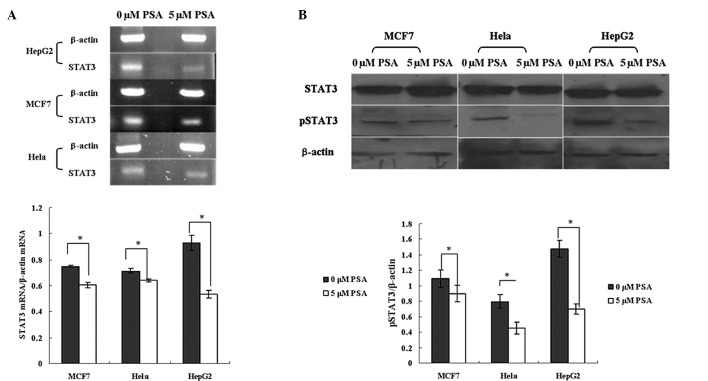
PSA reduces the level of STAT3 mRNA and pSTAT3 in HepG2, HeLa and MCF7 cells. Cells were treated with 5 μM of PSA for 48 h, then RT-PCR and western blot assay were performed to detect the level of mRNA and STAT3 protein. (A) Effect of 5 μM PSA on the expression of STAT3 mRNA. The histogram shows the ratio of STAT3 mRNA and β-actin mRNA, represented as the mean ± SD. n=3.(^*^P<0.05). (B) Effect of 5 μM PSA on the expression of STAT3 and pSTAT3 protein. The histogram shows the ratio of pSTAT3 and β-actin, represented as the mean ± SD (n=3). PSA, Prosapogenin A; STAT3, signal transducer and activator of transcription 3.

The expression of STAT3 mRNA and pSTAT3 in the HepG2, HeLa and MCF7 cells was significantly decreased by PSA treatment. The RT-PCR results showed that 5 μM PSA decreased the STAT3 mRNA level of the HepG2, MCF7 and HeLa cells by ~42, 19 and 10%, respectively (Fig. 3A). PSA did not significantly affect the expression of the STAT3 protein, but 5 μM PSA significantly decreased the level of pSTAT3 (Fig. 3B). The results of these experiments therefore indicate that the anticancer effect of PSA is mediated by the inhibition of the STAT3 pathway.

### Effect of PSA on the expression of the STAT3 target genes

The effect of PSA on the expression of the STAT3 target genes was analyzed with a Human STAT3-Regulated cDNA Plate Array kit. The results showed that 5 μM PSA downregulated the ratio of Bcl-2/Bax and the expression of survivin and glycoprotein 130 (gp130), and upregulated the expression of c-myc, C-reactive protein (CRP), cyclin E, glycogen synthase kinase 3β (GSK-3β), IL-10, oncostatin M (OSM), p21 and p27, subsequently promoting cell apoptosis. Therefore, PSA is able to promote cell apoptosis by inhibiting the STAT3 pathway.

### Effect of PSA on glycometabolism-related genes

**Figure 5:**
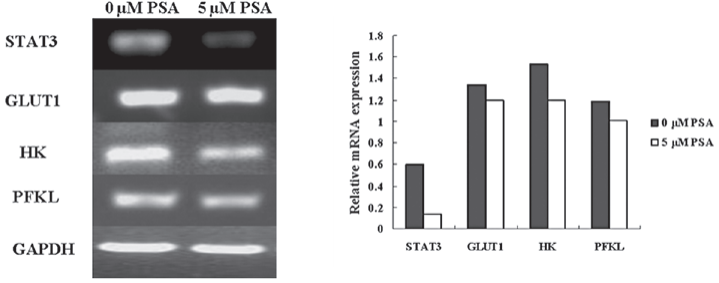
Expression of glycometabolism-related genes. MCF7 cells were treated either with or without 5 μM PSA for 48 h. RT-PCR detected the change in STAT3, GLUT1, HK and PFKL mRNA. PSA, Prosapogenin A; STAT3, signal transducer and activator of transcription 3; GLUT1, glucose transporter 1; HK, hexokinase; PFKL, phosphofructokinase.

Cancer cells utilize the glycolytic pathway to maintain cell growth even when adequate oxygen is present; this is known as the Warburg effect (17). Glycolysis inhibition is a potential therapeutic modality. The present study examined whether PSA affected the expression of the glycometabolism-related genes, glucose transporter 1 (GLUT1), hexokinase (HK) and phosphofructokinase (PFKL). The western blotting results showed that 5 μM PSA reduced the expression of the STAT3, GLUT1, HK and PFKL mRNA (Fig. 5). Therefore, the PSA-induced inhibition of the glycometabolic pathway may be another anticancer mechanism, and may be due to the downregulation of STAT3.

## Discussion

Chemotherapy remains one of the main therapeutic approaches for cancer patients. However, the cytotoxicity against normal cells limits the use of chemotherapeutic drugs. It is therefore necessary to develop anticancer agents with minimal side-effects. Traditional Chinese medicine has relatively fewer side-effects and has been used to treat various diseases in clinical medicine, including cancer, for numerous years (18–22). As a well-known traditional Chinese medicine, *Veratrum* has shown significant anticancer effects in a variety of carcinomas (14–16).

The transcription factor STAT3 is, as an oncogene, crucial for the regulation of cancer cell apoptosis and proliferation. The phosphorylation of STAT3 at tyrosine 705 results in its homodimerization, nuclear translocation and DNA binding, which consequently regulates the expression of various critical genes involved in cell proliferation and apoptosis (23–25). The constitutive activation of STAT3 results in the development of various types of cancer. Therefore, the regulation of the STAT3 pathway and the expression of its target genes has been a promising area for the development of anticancer therapies.

The present study demonstrated a novel anticancer effect of PSA from *Veratrum* on cervical carcinoma (HeLa), hepatocellular carcinoma (HepG2) and breast adenocarcinoma (MCF-7) cells, and provided data to indicate its possible mechanism. Several new points may consequently be reported. First, PSA was demonstrated to cause a concentration- and time-dependent reduction in cancer cell survival. PSA caused a smaller reduction in the survival of normal, i.e. non-cancer, cells. Second, PSA was identified to be able to induce a large increase in the number of apoptotic cells, at the same time as a clear cell cycle arrest in the G_2_/M phase. Third, PSA was shown to inhibit the expression of STAT3 mRNA and the phosphorylation of the STAT3 protein. Fourth, PSA was demonstrated to largely suppress the STAT3 downstream genes, Bcl-2/Bax, survivin and gp130, and cause clear elevation in the expression of c-myc, CRP, cyclin E, GSK-3β, IL-10, OSM, p21 and p27, subsequently promoting cell apoptosis. Finally, PSA regulated the expression of the glycometabolism-related genes to inhibit cell growth and promote cell apoptosis.

In conclusion, PSA exhibits clear anticancer cell activity *in vitro*. PSA is able to block the STAT3 pathway and modulate the expression of glycometabolism-related genes, resulting in a reduction in cell proliferation and an increase in cell apoptosis.

## Figures and Tables

**Figure 4:**
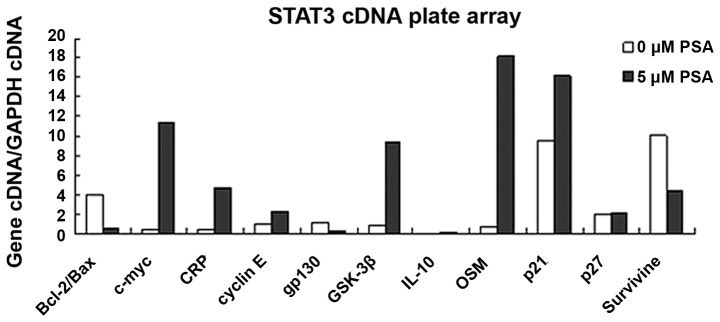
STAT3 regulated cDNA plate assay for analysis of PSA-induced STAT3 target genes. HepG2 cells were treated either with or without 5 μM PSA for 48 h. RNAs were subjected to STAT3 cDNA plate array assay. PSA, Prosapogenin A; STAT3, signal transducer and activator of transcription 3; CRP, C-reactive protein; GSK-3β, glycogen synthase kinase 3β; OSM, oncostatin M.
